# 18F-FDG PET/CT Findings to Improve Confidence in Distinguishing Lung External Beam Radiotherapy Side Effects

**DOI:** 10.3390/life15091392

**Published:** 2025-09-02

**Authors:** Dino Rubini, Valerio Nardone, Corinna Altini, Claudia Battisti, Cristina Ferrari, Alfonso Reginelli, Federico Gagliardi, Giuseppe Rubini, Salvatore Cappabianca

**Affiliations:** 1 Department of Precision Medicine, University of Campania “L. Vanvitelli”, 80131 Naples, Italy; valerio.nardone@unicampania.it (V.N.); alfonso.reginelli@unicampania.it (A.R.); fedegagliardi@hotmail.it (F.G.); salvatore.cappabianca@unicampania.it (S.C.); 2 Nuclear Medicine Unit, Interdisciplinary Department of Medicine, University “Aldo Moro” Bari, 70124 Bari, Italy; corinna.altini@hotmail.it (C.A.); claudiabattisti93@gmail.com (C.B.); ferrari_cristina@inwind.it (C.F.); giuseppe.rubini@uniba.it (G.R.)

**Keywords:** lung cancer, radiotherapy, EBRT, 18F-FDG PET/CT, qualitative analysis, manually semiquantitative analysis

## Abstract

Modern external beam radiotherapy (EBRT) on lung cancer improved dose distribution thanks to advanced dose calculation algorithms, but side effects and relapses can occur in any case onset. Differential diagnosis of relapses and side effects is difficult, and when computed tomography (CT) is uncertain 18-fluorodeoxyglucose-positron emission tomography/computed tomography (18F-FDG-PET/CT) can support the diagnosis, even if it can also be difficult to construe. The aim of this retrospective analysis was to evaluate 18F-FDG PET/CT qualitative patterns and semiquantitative parameters, both automatic and preceded by physicians, in interpreting lung lesions in the radiotherapy (RT) lung irradiation field. In total, 94 patients (pts) submitted to EBRT (3 months before) for stage II lung cancer were included (74 men, 20 women, mean age of 68 years old, range of 49–84 years old). CT scans were performed on pts, which showed lung lesions in the RT field. 18F-FDG-PET/CT scans were analyzed qualitatively as negative or positive, and the presence of the lung area with a high 18F-FDG uptake pattern was distinguished as the following: focal/wide, deep/shade, or homogeneous/inhomogeneous. Furthermore, the following semiquantitative parameters were collected: gSUVmax (global standardized uptake value max), MTV (tumor metabolic volume), metabolic spatial distribution (MSD) = proximal SUVmax/distal SUVmax, and intratumoral difference in spatial distribution (IDSD%) = [distal SUVmax/proximal SUVmax] × 100. 18F-FDG PET/CT was related to the pts’ outcome (biopsy and/or clinical–instrumental follow-up): positive for lung relapse, negative if the lesions were phlogistic. The following diagnostic performance parameters of 18F-FDG PET/CT were calculated: sensitivity (Sens), specificity (Spec), diagnostic accuracy (DA), positive predictive value (PPV), and negative predictive value (NPV). Qualitative variables were compared by Chi-squared test, while for semiquantitative parameters Student’s *t*-test was applied; *p* < 0.05 was considered statistically significant. Statistics tests were performed with MedCalc V.22.018 ©2024. In 76/94 (80.8%) pts, 18F-FDG uptake was higher compared to the background; in 18/94 (19.2%) no high 18F-FDG uptake areas were detected. Outcome was positive for lung relapse in 49/94 pts, while negative in 45/94, with disease prevalence of 52.13% (95%CI = 41.57–62.54%). In the 18/94 pts without high 18F-FDG uptake, the outcome was negative for lung relapse. In 49/76 pts with higher 18F-FDG uptake, the outcome confirmed the presence of relapse, while in 27/76 the lesion was phlogistic. Results about the Sens, Spec, DA, PPV, and NPV (95%CI) were, respectively: 100% (92.75–100%), 40% (25.7–55.67%), 71.28% (61.02–80.14%), 64.47% (58.84–69.73%), and 100% (81.47–100%). Chi-square test showed significant statistical difference between the positive and negative outcome for patterns focal/wide (*p* = 0.02) and deep/shade (*p* < 0.00001). A total of 35/49 (71.4%) pts with lung relapse had a focal lesion and 15/27 (55.6%) with phlogosis had a wide pattern. A total of 34/49 (69.4%) pts with lung relapse had a deep pattern and 25/27 (92.6%) with lung phlogosis had the shade one. Significant difference was observed in evaluating the three patterns (*p* = 0.00007), with prevalence of “focal/deep/homogeneous” patterns in lung relapse and “wide/shade/inhomogeneous” in phlogosis. gSUVmax, MTV, MSD, and IDSD% were in the following order: in the 76 pts, 5.63 (1.4–24.7), 42.49 (4.94–193), 3.61 (1–5.54), and 70.7% (18–100%); in the 49/76 true positive pts, 6.93 (1.5–24.7), 35.28 (4.94–85.99), 3.30 (1.05–5.54), and (18–95%); in the 27/76 false positive pts, 3.27 (1.4–19.2), 38.37 (4.94–193), 1.57 (1–2.13), and 78.6% (4.7–100%). The difference was statistically significant only for MSD (t = 2.779; *p* = 0.0069) and IDSD% (t = 2.769; *p* = 0.0071). 18F-FDG-PET/CT confirms its high sensitivity and NPV in evaluating lung lesions after RT. To improve physician confidence in interpreting lung 18F-FDG uptake without further support, MSD and IDSD% could be considered. Heterogeneity of lung lesions, especially in radiotreated tissue, can be turned from a drawback to a resource and analyzed for differentiating relapses from EBRT side effects. Considering the calculation of semiquantitative parameters that require “human intelligence”, even if slightly more time-consuming, can improve the nuclear physician’s confidence in interpreting 18F-FDG PET/CT images.

## 1. Introduction

Lung cancer is the leading cause of cancer-related mortality in both men and women, with a global incidence of 11.6% and a mortality rate of 18.4% [1].

Non-small-cell lung cancer (NSCLC) accounts for over 80% of lung cancer cases, approximately 55% of which present with distant metastases at diagnosis, while about 30% manifest as less invasive disease, such as Stage II [2].

Treatment options for NSCLC are diverse and may be employed alone or in combination, depending on disease stage and characteristics, as well as patient factors including age, operability, and drug sensitivity [3].

Radiation therapy (RT) represents one of the most effective treatment modalities, utilized either as monotherapy or combined with conventional approaches such as surgery, chemotherapy, and immunotherapy [4].

Recent advances in RT technology have enabled the development of multiple sophisticated techniques, resulting in improved local tumor control and enhanced patient survival [5].

External beam radiation therapy (EBRT) is a broad yet effective modality encompassing various techniques with specific advances and adaptations tailored to tumor characteristics (e.g., intensity-modulated radiation therapy, stereotactic body radiation therapy, image-guided radiation therapy, volumetric modulated arc therapy, proton therapy, heavy ion therapy) [5].

Modern EBRT treatment has demonstrated optimal outcomes due to improvements in dose calculation algorithms, delivery methods, and dose distribution, enabling superior tumor targeting, enhanced tumor delineation, and reduced irradiation of normal tissues. Nevertheless, collateral damage to normal tissues, both inflammatory and fibrotic, can still occur, and tumor recurrence at the treated site remains possible [6].

Therefore, timely differentiation between malignant pulmonary lesions and EBRT-induced reactions is essential. Currently, thoracic computed tomography (CT) is the imaging modality of choice, although equivocal cases may arise [7].

Positron emission tomography combined with 2-deoxy-2-[18F]fluoro-D-glucose/computed tomography (18F-FDG PET/CT) is a valuable diagnostic tool in the evaluation of NSCLC, based on increased glucose metabolism in malignant cells [8].

18F-FDG PET/CT is widely employed for differential diagnosis of pulmonary lesions for both primary and post-EBRT changes; however, 18F-FDG is not tumor-specific, and, as with CT, indeterminate lesions can be encountered [9,10].

To support qualitative interpretation of 18F-FDG PET/CT, semiquantitative parameters are analyzed, among which the maximum standardized uptake value (SUVmax) is the most commonly evaluated due to its reproducibility. Nonetheless, SUVmax has limitations in differential diagnosis because overlapping values have been observed between pulmonary recurrences and EBRT-induced side effects in a substantial proportion of patients [10].

Previous studies have reported a specificity of 18F-FDG PET/CT for malignant pulmonary nodule diagnosis ranging from 13% to 89%, with lower specificity in previously treated lungs [11].

Metabolic tumor volume (MTV) is a semiquantitative parameter that may provide prognostic information regarding treatment response in lung cancer, although its role has not yet been fully validated [12].

Hence, there is a need for alternative diagnostic parameters to enhance the diagnostic accuracy of pulmonary lesions.

A characteristic feature of 18F-FDG distribution in malignant lung tumors is heterogeneity, and its evaluation could be useful in distinguishing these from EBRT-induced effects [10].

18F-FDG PET/CT demonstrates a low positive predictive value (PPV) in differentiating recurrent tumors from post-radiotherapy inflammation, which may persist up to six months following treatment, although it performs better in distinguishing these from fibrosis [13].

Interpretation of 18F-FDG PET/CT images in routine clinical practice primarily relies on visual criteria, while quantitative parameters provide complementary information [14].

Image description using semantic features represents the standard clinical approach. In lung cancer, 18F-FDG PET/CT images are assessed considering the presence of areas with altered 18F-FDG uptake and describing their number, size, shape, and distribution. These features are globally accepted criteria for evaluating therapeutic response, despite some limitations [15].

Nuclear medicine physicians typically consider patterns such as lesion size, intensity of 18F-FDG uptake, and distribution. Lesion size generally does not correlate with lesion nature, as even small nodules may be malignant. Local recurrence tends to be more focal, whereas inflammation exhibits a more diffuse pattern [13].

The intensity of 18F-FDG uptake is more indicative of malignancy as it tends to be higher in neoplastic cells with elevated glucose metabolism, although activated inflammatory cells can also demonstrate increased metabolism. Regarding uptake distribution within the lesion, malignant lesions are generally more homogeneous. The intensity and distribution of uptake reflect the level of glucose metabolism, which correlates with necrosis, angiogenesis, cellular proliferation, blood flow, and hypoxia: all of these variables contribute to metabolic heterogeneity. Studies have demonstrated that blood supply is greater in the proximal region of lung tumors, resulting in higher 18F-FDG uptake; consequently, malignant lesions appear more homogeneous, whereas in pulmonary inflammation blood supply is predominantly distal, producing the opposite effect [12].

However, none of these patterns when evaluated individually are definitive for differential diagnosis, especially in previously treated lung tissue.

Similarly, SUVmax is influenced by heterogeneity but remains the most widely used semiquantitative parameter to assess 18F-FDG uptake intensity, despite a significant overlap of SUVs between benign and malignant lesions which limits specificity. For example, malignant lesions such as bronchioloalveolar carcinoma may exhibit low SUV [16], while inflammatory lesions can show high SUV [7]. Currently, no universally accepted cut-off values exist to distinguish malignant pulmonary lesions, considering that factors like blood glucose levels and PET/CT technical characteristics influence SUV measurements [14].

Few studies, such as that by Lin et al. (2020), have reported statistically higher SUVmax values in malignant nodules compared to benign ones when analyzing untreated lungs [11].

The aim of this study was to evaluate the utility of qualitative patterns and semiquantitative parameters, calculated both automatically and manually, for the analysis of 18F-FDG PET/CT images based on the spatial distribution of 18F-FDG, to assist clinicians in differentiating pulmonary recurrences from EBRT-induced inflammatory effects.

## 2. Materials and Methods

### 2.1. Patients

From January 2017 to December 2022, a total of 94 patients who underwent 18F-FDG PET/CT for suspected malignant pulmonary recurrence were included in this retrospective study. The demographic characteristics of the patients were as follows: 74 (78.7%) men and 20 (21.3%) women; mean age 68 years; median age 70 years; and range 49–84 years.

All patients had lung adenocarcinoma and were initially classified as stage II according to the international NCCN guidelines [2] and underwent EBRT for NSCLC. The patients were included in stages IIB and IIA, and therefore, lymph nodes and N1 cases were also studied.

All patients underwent chest computed tomography (CT) at least 3 months after completing RT and 18F-FDG PET/CT within 6 weeks of the CT, thus shortly after the 3-month period following the completion of radiotherapy.

The choice of EBRT modality and dose fractionation scheme, as well as previous surgical interventions, varied according to the clinical and pathological characteristics of each patient.

The inclusion criteria for subjects were as follows: (I) stage II primary lung cancer previously treated with EBRT; (II) CT describing a lesion in the EBRT area; (III) availability of follow-up data. The exclusion criteria were as follows: (I) common exclusion criteria for exposure to ionizing radiation; (II) history of other synchronous or metachronous neoplasms; (III) evidence of metastasis in sites other than the treated pulmonary area, both on CT and 18F-FDG PET/CT; (IV) motion artifacts.

For all patients, the definitive diagnosis was made through pathological analysis or at least 1 year of follow-up with chest CT.

The study was conducted in accordance with the Declaration of Helsinki. All patients signed informed consent for the use of their data for scientific purposes in an anonymous form before undergoing 18F-FDG PET/CT. Our institutional ethics committee does not require approval for the review of patient records.

The study protocol was approved by the relevant ethical committees. The study was conducted in accordance with ICH guidelines for good clinical practices and the Declaration of Helsinki.

### 2.2. 18F-FDG PET/CT Procedure

Before administration of 18F-FDG, all patients underwent fasting for at least 6 h and had a capillary blood glucose level of less than 160 mg/mL. To avoid muscle artifacts, patients were instructed not to perform physical activity before the examination. After intravenous administration of 4.6 MBq/kg of 18F-FDG, patients were hydrated by drinking 500 mL of water and urinating if necessary, and imaging acquisition occurred 50 min later.

Images were acquired using a combined PET/CT system (Discovery LSA, GE Healthcare, Waukesha, WI, USA), which integrates a PET scanner (Advance NXI) with a 16-slice CT scanner (LightSpeed Plus).

The scan was performed from the external ear to the root of the thigh, with patients in the supine position and hands above the head. A head scan was also performed.

The CT acquisition parameters were as follows: 340 mA (auto), 120 kV, slice thickness 3.75 mm, tube rotation time 0.8 ms, and collimation FOV 50 cm. The CT images were reconstructed using filtered back-projection. The CT data were used for attenuation correction of the PET scans, which were performed immediately after CT image acquisition. CT scans were obtained without contrast media administration. The PET acquisition was reconstructed with a 128 × 128 matrix, iterative reconstruction algorithm with maximum likelihood expectation (two iterations, 28 subsets), 8 mm Gaussian filter, and a 50 cm FOV.

### 2.3. Image Analysis

Image analysis was performed on the Xeleris™ workstation (GE Healthcare, Waukesha, WI, USA) for reconstruction of PET, CT, and PET/CT fusion images.

A nuclear medicine physician selected the 18F-FDG PET/CT scans based on the inclusion and exclusion criteria, and two nuclear medicine physicians independently analyzed the data in a blinded manner; an additional nuclear medicine physician, unaware of the patient’s history, was involved in cases of discrepancies.

The lung area treated with EBRT was identified in the co-registered CT images, and 18F-FDG uptake was visually assessed. In the case of increased uptake compared to background, it was considered positive; in the absence of uptake, the examination was considered negative.

In the case of 18F-FDG uptake, the pattern was classified as follows: (I) focal/wide; (II) deep/shadow; (III) homogeneous/heterogeneous.

For the semiquantitative analysis, volumes of interest (VOIs) were semi-automatically drawn in the areas of high 18F-FDG uptake consistent with the patient’s history of cancer and modulated according to the parameter to be collected by nuclear medicine experts.

The standardized uptake values (SUVs) were calculated using the maximum activity values within each VOI on the axial slices with the highest radioactive concentration, normalized to the injected dose and the patient’s body weight; VOIs were manually delineated to encompass the entire area of lung parenchymal alteration, including portions of altered parenchyma without PET uptake but spatially contiguous to the uptake region.

The semiquantitative parameters collected were global SUVmax (gSUVmax) and tumor metabolic volume (MTV) across the entire lung area with 18F-FDG uptake; proximal SUVmax (pSUVmax) in the visually most intense area of 18F-FDG uptake; and distal SUVmax (dSUVmax) in the visually least intense area of uptake. The parameter called metabolic spatial distribution (MSD) was derived from the ratio of proximal SUVmax to distal SUVmax (MSD = pSUVmax/dSUVmax). The parameter called intratumoral difference in spatial distribution (IDSD) was calculated as the percentage of the ratio between distal SUVmax and proximal SUVmax (IDSD% = [dSUVmax/pSUVmax] × 100).

### 2.4. Statistical Analysis

The 18F-FDG PET/CT was correlated with the patient’s definitive diagnosis (biopsy and/or clinical–instrumental follow-up): positive for pulmonary recurrence and negative for inflammation.

The following diagnostic performance parameters for 18F-FDG PET/CT were calculated: sensitivity (Sens), specificity (Spec), diagnostic accuracy (DA), positive predictive value (PPV), and negative predictive value (NPV).

Qualitative variables were compared using the Chi-square test, while Student’s *t*-test was applied to semiquantitative parameters: *p* < 0.05 was considered statistically significant. Statistical tests were performed with MedCalc V.22.018 ©2024.

## 3. Results

### 3.1. 18F-FDG Diagnostic Performances

In 76/94 (80.8%) pts, 18F-FDG uptake was higher compared to the background; in 18/94 (19.2%), no higher 18F-FDG uptake was detected.

Outcome was positive for lung relapse in 49/94 pts, while it was negative in 45/94, with disease prevalence of 52.13% (95%CI = 41.57–62.54%). In the 18/94 pts without higher 18F-FDG uptake, the outcome was negative for lung relapse. In 49/76 pts with higher 18F-FDG uptake, the outcome confirmed the presence of relapse, while in 27/76 the lesion was phlogistic. Results about the Sens, Spec, DA, PPV, and NPV (95%CI) were, respectively, 100% (92.75–100%), 40% (25.7–55.67%), 71.28% (61.02–80.14%), 64.47% (58.84–69.73%), and 100% (81.47–100%).

### 3.2. Qualitative Assessment

Distribution of the lesion patterns in the 76/94 patients with high 18F-FDG uptake were as follows: focal 47/76 (61.8%), wide 29/76 (38,2%); deep 36/76 (47.4%), shade 40/76 (52.6%); and homogeneous 46/76 (60.5%), inhomogeneous 30/76 (39.5%). These data are reported on Table 1.

**Figure 1:**
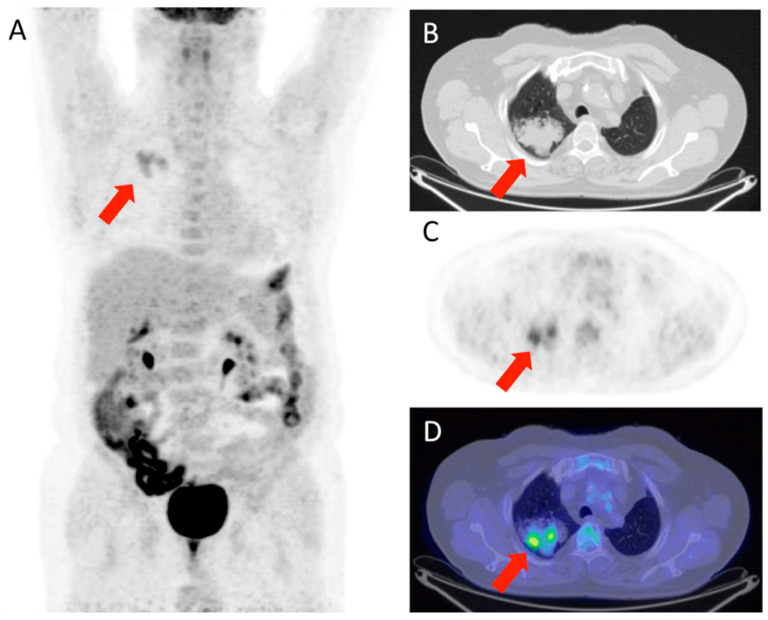
Patient with lung relapse. 18F-FDG PET/CT was interpreted as positive for 18F-FDG uptake as MIP (**A**), transaxial CT (**B**), PET (**C**), and fused (**D**) images showed 18F-FDG uptake described as focal (with two foci), deep, and inhomogeneous. The semiquantitative parameters were gSUVmax 3.6, MTV 17,41, pSUVmax 3.4, dSUVmax 1.18, MSD 2.87, and IDSD% 34.7%.

**Figure 2:**
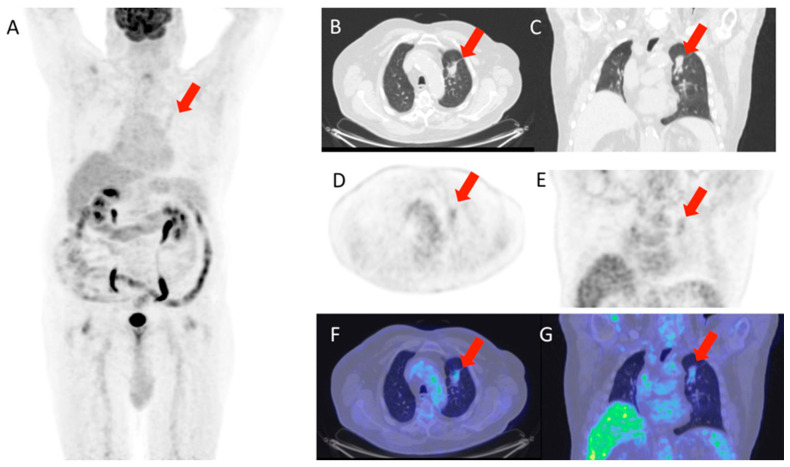
Patient with lung phlogosis. The images shown (**A**–**G**) are sequences from the same PET/CT of a patient under consideration; specifically, (**A**) is a total body scan, (**B**,**C**) are axial and coronal CT scans of the chest; (**D**,**E**) are the corresponding PET scans in axial and coronal views; (**F**,**G**) show the fusion of CT and PET to illustrate the station level of the region of interest.

Figure 1 and Figure 2 show, respectively, examples of patients with lung relapse and a patient with phlogistic reaction to EBRT. In detail, Figure 1 shows images of a patient (male, 68 years old) with lung recurrence; PET/CT images with 18F-FDG are shown of a patient diagnosed with right lung adenocarcinoma who underwent surgery two years earlier and subsequently underwent radiotherapy for right lung recurrence, and the contrast-enhanced CT scan (Figure 1B) performed three months after the end of radiotherapy showed extensive pulmonary thickening in the right lung. Figure 2 shows an example of a patient with pulmonary inflammation: it shows the 18F-FDG PET/CT scan of an 82-year-old male patient diagnosed with left lung adenocarcinoma who underwent RT. The chest CT scan performed 6 months after the end of RT showed a lesion in the left lung with spiculated margins and pleural connection streaks. The ^18F-FDG PET/CT was interpreted as positive for ^18F-FDG uptake; MIP images (A), axial and coronal CT (B, C), PET (D, E), and fusion images (F, G) described the uptake of ^18F-FDG as focal, deep, and homogeneous. The semiquantitative parameters were as follows: gSUVmax 2.3, MTV 47.60, pSUVmax 2.1, dSUVmax 1.9, MSD 1.1, and IDSD% 90.5%. The lesion was initially interpreted as a pulmonary recurrence; however, follow-up showed that it was a side effect of EBRT as it resolved within 6 months.

The lesion was interpreted as a lung relapse, which was confirmed by biopsy performed on the focus with highest uptake (SUVmax 3.4).

Chi-square test showed difference statistical significance between the positive and negative outcomes for patterns focal/wide (*p* = 0.02) and deep/shade (*p* < 0.00001). A total of 35/49 (71.4%) pts with lung relapse had focal lesions and 15/27 (55.6%) with phlogosis had wide patterns. A total of 34/49 (69.4%) pts with lung relapse had deep patterns, and 25/27 (92.6%) with lung phlogosis had shade ones. Significant difference was observed in evaluation of the three combined patterns (*p* = 0.00007), with prevalence of “focal/deep/homogeneous” patterns in lung relapse and “wide/shade/inhomogeneous” in phlogosis.

### 3.3. Semiquantitative Assessment

Semiquantitative assessment was performed only on the 76 patients with high 18F-FDG uptake. The difference was statistically significant only for the manually calculated parameters MSD (t = 2.779; *p* = 0.0069) and IDSD% (t = 2.769; *p* = 0.0071). Related results are reported in Table 2.

## 4. Discussion

Pulmonary external beam radiotherapy (EBRT) may be followed by acute adverse effects predominantly affecting rapidly renewing tissues, typically causing inflammation that is generally reversible, although healing can be prolonged. Late adverse effects of EBRT include fibrosis, resulting from proliferation of surviving fibroblasts stimulated by released growth factors, and recurrences, caused by survival of residual tumor cells or damaged healthy cells stimulated by the surrounding microenvironment [6].

Recurrence in the treated lung represents the main clinical challenge and usually occurs within 2 years after treatment, a period during which other adverse effects are also highly likely, complicating differential diagnosis [13].

According to guidelines from NCCN, the European Society for Medical Oncology (ESMO), and the European Society for Radiotherapy and Oncology (ESTRO-ACROP), radiological follow-up should include chest CT and 18F-FDG PET/CT in cases of equivocal lesions [2,16,17].

Currently, 18F-FDG PET/CT is considered a cornerstone in the evaluation of numerous oncologic diseases across various diagnostic and therapeutic stages, and it has also proven useful in the assessment of non-oncologic and inflammatory diseases that exhibit high 18F-FDG uptake [9].

The uptake of 18F-FDG in both inflammatory and oncologic conditions shares a similar mechanism mediated by glucose transporters GLUT1-5. Specifically, in active inflammation, increased 18F-FDG uptake is due to high expression of GLUT1 in activated leukocytes, macrophages, and T lymphocytes [11].

These mechanisms explain why 18F-FDG PET/CT demonstrates high sensitivity but only moderate specificity in malignancy detection [7]. In our study, sensitivity was optimal (100%), but specificity was relatively low (40%) compared to published literature [11]; conversely, our positive predictive value (PPV) (64.47%) aligns with the literature data [13].

As previously mentioned, clinical interpretation of 18F-FDG PET/CT images is primarily based on visual criteria and complemented by quantitative parameters, and semantic features assessed include number, size, shape, and distribution of areas with altered uptake. Notably, lesion width poorly distinguishes between malignant and benign lesions, whereas uptake intensity and homogeneity are more indicative—malignant lesions tend to have more intense and homogeneously distributed uptake compared to inflammatory lesions, which exhibit a more diffuse pattern. However, no single pattern is sufficient for a definitive diagnosis, especially in previously treated lungs.

Consistent with literature data [12,13,15], our study found statistically significant differences only when analyzing the combination of three patterns, where extensive areas of 18F-FDG uptake, shadowing, and inhomogeneity are more indicative of inflammatory adverse effects from EBRT.

As described in the introduction, SUVmax—the most commonly used semiquantitative parameter to evaluate 18F-FDG uptake intensity—is influenced by heterogeneity and has specificity limitations due to overlaps between benign and malignant lesions. No universally accepted cut-off values exist because factors such as blood glucose levels and PET/CT technical characteristics also influence SUV. In our study, no statistically significant difference was observed between malignancy and EBRT effects, although the sample size was small.

Metabolic tumor volume (MTV) is another semiquantitative parameter proposed for assessing tumor burden, but it is not yet widely applied clinically due to poor reproducibility across institutions [14].

To our knowledge, no published studies have differentiated pulmonary recurrences from EBRT side effects; our study also did not show a diagnostic role for these parameters in this regard.

Other methods based on radiomics have been proposed for evaluating neoplastic lesions. Radiomics has proven valuable for assessing radiotherapy response and side effects and has shown some predictive capability for recurrence. Despite promising results, radiomics remains a data-intensive approach that is not yet validated; its application requires large medical imaging databases, standardized imaging procedures, and data processing guidelines [15,18].

Considering radiomics as a potential future direction, alternative semantic features have been proposed for lung cancer analysis that do not require costly or specialized software but can be performed with common image visualization tools.

Lin et al. evaluated the role of a parameter called V-FMSD (visual assessment metabolic spatial distribution) to differentiate benign and malignant pulmonary nodules in untreated lungs by characterizing RAD (relative activity distribution), which was developed from analysis of metabolic heterogeneity in solitary pulmonary nodules (SPNs) [11].

This method relies on the visual assessment of proximal and distal lesion regions, assigning scores from one to five for each area. Results showed that nodules with greater differences between proximal and distal regions were mostly malignant, with malignant nodules showing higher 18F-FDG uptake proximally than distally. It is a simple clinical method but operator-dependent [11].

Previously, Liang et al. reported that RAD was more sensitive and specific than SUVmax in distinguishing benign from malignant nodules, with similar sensitivity and accuracy to nuclear medicine physicians’ visual assessment. It should be noted that analyses were performed on untreated pulmonary nodules, many of which were small [7].

Inspired by RAD evaluation and the concept of heterogeneity, we developed two parameters (MSD and IDSD%) to aid in the interpretation of PET images of pulmonary nodules. Unlike previous methods, ours requires only two parameters (pSUVmax and gSUVmax) and simple calculations to evaluate distribution differences within lesions without analytical software.

To our knowledge, our study is the first to propose MSD and IDSD% parameters and the first conducted on lungs treated with radiotherapy. Results showed statistically significant differences for both parameters in distinguishing secondary lesions from inflammatory reactions.

Strengths of the MSD and IDSD% methods include simplicity and ease of clinical application. Therefore, V-FMSD can be considered a new routine diagnostic adjunct for evaluating indeterminate pulmonary nodules and masses with high 18F-FDG uptake.

The main limitation of this study is its retrospective nature and small patient cohort, though patients were strictly selected to minimize confounding factors such as diabetes and active smoking.

Further prospective studies with larger, multicenter cohorts are needed to confirm and generalize these findings.

## 5. Conclusions

18F-FDG-PET/CT confirms its high sensitivity and NPV in evaluating lung lesion after RT. Heterogeneity of lung lesion, especially in radiotreated tissue, can be turned from a drawback to a resource and analyzed for differentiating relapses from EBRT side effects. MSD and IDSD% are simple semiquantitative parameters that can support differential diagnosis of lung relapse and EBRT lung side effects.

This study indicates that the calculation of semiquantitative parameters such as MSD and IDSD%, which requires “human intelligence”, can improve the nuclear physician confidence in interpreting 18F-FDG PET/CT images, even if it is a little more time consuming.

## Figures and Tables

**Table 1:** Distribution of the lesion patterns in the 76/94 patients with high 18F-FDG uptake.

Lesion Patterns	Number (%)
Focal	47 (61.8%)
Wide	29 (38.2%)
Deep	36 (47.4%)
Shade	40 (52.6%)
Homogeneous	46 (60.5%)
Inhomegeneous	30 (39.5%)

**Table 2:** The calculated average value along with the min and max values, given in parenthesis, for the gSUVmax (global SUVmax), MTV (tumor metabolic volume), MSD (metabolic spatial distribution), and IDSD% (intratumoral difference in spatial distribution) of the 76 patients.

	76 Patients with High 18F-FDG Uptake	49/76 True Positive Patients	27 False Positive Patients
gSUVmax	5.63 (1.4–24.7)	6.93 (1.5–24.7)	3.27 (1.4–19.2)
MTV	42.49 (4.94–193)	35.28 (4.94–85.99)	38.37 (4.94–193)
MSD	3.61 (1–5.54)	3.30 (1.05–5.54)	1.57 (1–2.13)
IDSD%	70.7% (18–100%)	35.28 (4.94–85.99)	78.6% (4.7–100%)

## Data Availability

The original contributions presented in this study are included in the article. Further inquiries can be directed to the corresponding author.
